# Supplementary figures and images for: In vitro evaluation of PEI-coated microbubble– neutrophil conjugates for ultrasound-guided cell delivery
Source: Front Immunol. 2026 Feb 12;17:1743853. doi: 10.3389/fimmu.2026.1743853 (PMC12936041; doi:10.3389/fimmu.2026.1743853)

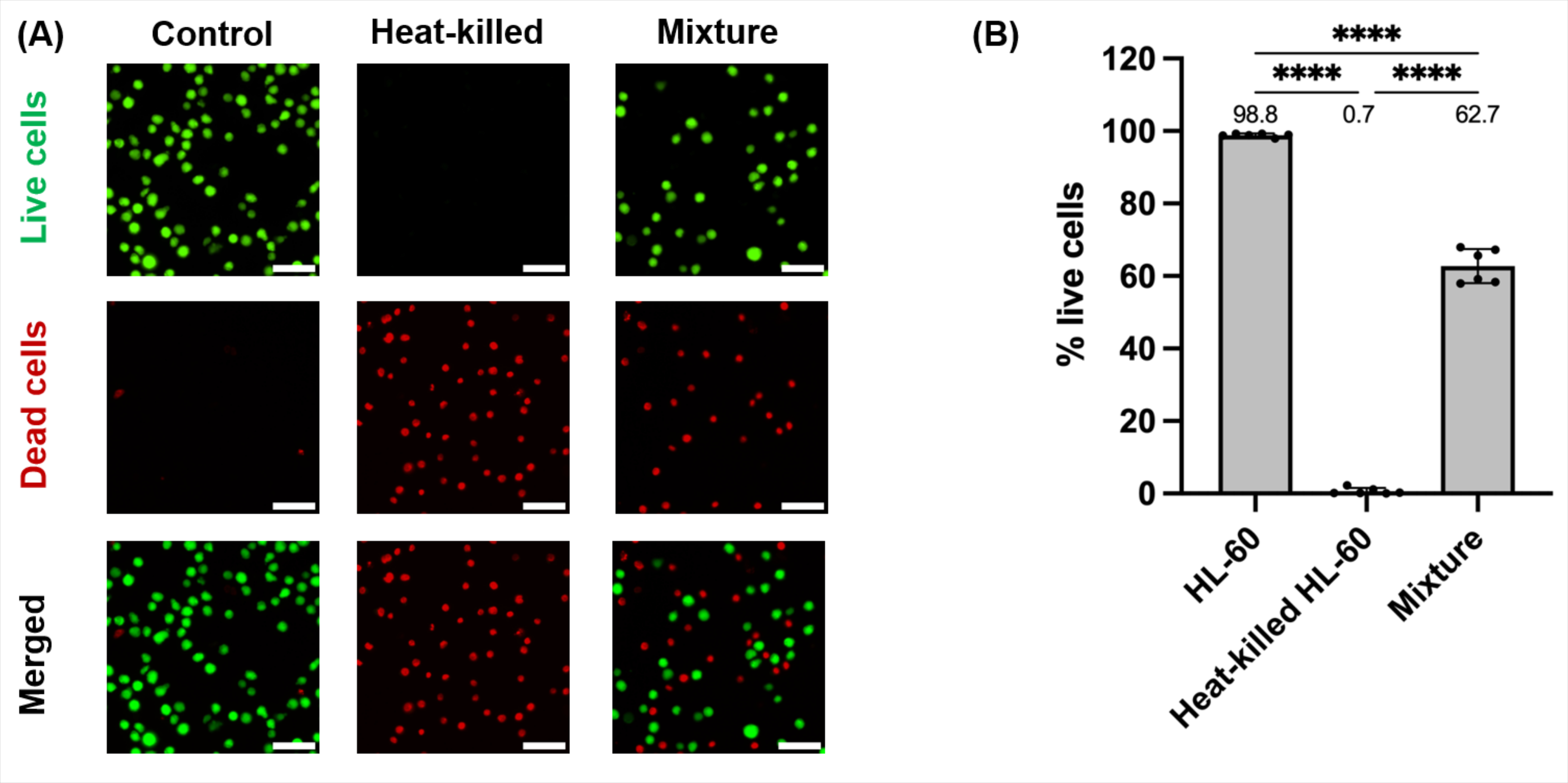

Supplement: Supplementary file 1 [file Image1.jpeg]
